# PositiveLinks: A Mobile Health Intervention for Retention in HIV Care and Clinical Outcomes with 12-Month Follow-Up

**DOI:** 10.1089/apc.2017.0303

**Published:** 2018-06-01

**Authors:** Rebecca Dillingham, Karen Ingersoll, Tabor E. Flickinger, Ava Lena Waldman, Marika Grabowski, Colleen Laurence, Erin Wispelwey, George Reynolds, Mark Conaway, Wendy F. Cohn

**Affiliations:** ^1^ ^1^Department of Medicine, University of Virginia School of Medicine, Charlottesville, Virginia.; ^2^ Department of Psychiatry and Neurobehavioral Sciences, University of Virginia School of Medicine, Charlottesville, Virginia.; ^3^ Health Decision Technologies, Oakland, California.; ^4^ Department of Public Health Sciences, University of Virginia School of Medicine, Charlottesville, Virginia.

**Keywords:** mobile health, smartphone app, retention in care, HIV/AIDS, positive links

## Abstract

Mobile health interventions may help People Living with HIV (PLWH) improve engagement in care. We designed and piloted PositiveLinks, a clinic-affiliated mobile intervention for PLWH, and assessed longitudinal impact on retention in care and viral suppression. The program was based at an academic Ryan White Clinic serving a nonurban population in Central Virginia. The PL intervention included a smartphone app that connected participants to clinic staff and provided educational resources, daily queries of stress, mood and medication adherence, weekly quizzes, appointment reminders, and a virtual support group. Outcomes were analyzed using McNemar's tests for HRSA-1, visit constancy, and viral suppression and nonparametric Wilcoxon signed-rank tests for CD4 counts and viral loads. Of 77 participants, 63% were male, 49% black non-Hispanic, and 72% below the federal poverty level. Participants' achievement of a retention in care benchmark (HRSA-1) increased from 51% at baseline to 88% at 6 months (*p* < 0.0001) and 81% at 12 months (*p* = 0.0003). Visit constancy improved from baseline to 6 months (*p* = 0.016) and 12 months (*p* = 0.0004). Participants' mean CD4 counts increased from baseline to 6 months (*p* = 0.0007) and 12 months (*p* = 0.0005). The percentage of participants with suppressed viral loads increased from 47% at baseline to 87% at 6 months (*p* < 0.0001) and 79% at 12 months (*p* = 0.0007). This study is one of the first to demonstrate that a mobile health intervention can have a positive impact on retention in care and clinical outcomes for vulnerable PLWH. Next steps include integration with clinical practice and dissemination.

## Introduction

HIV is a chronic, manageable disease, but only if patients engage with and stay in care. The 90-90-90 UNAIDS proposal sets global recommendations that by 2020, 90% of HIV-infected persons know their status, 90% of those who are HIV positive receive antiretroviral therapy (ART), and 90% of those receiving ART have viral suppression.^1^ Medication adherence is referred to as the “Plus” in the 90-90-90-Plus global challenge, emphasizing the importance of ART adherence in achieving viral suppression.^2^ Many gaps remain in meeting these goals. Twenty percent of People Living with HIV (PLWH) never establish primary HIV care after their initial visit and half of established patients have gaps in care.^3^ Missed appointments for HIV care are associated with lower likelihood of achieving viral suppression, transmission risk behaviors, and mortality.^4,5^ Many factors can contribute to poor engagement with care, including age, gender, socioeconomic status, comorbidities, and unmet psychosocial needs.^6,7^ Systemic issues, such as patient trust in physicians and healthcare institutions, can also influence retention in care.^8^ Racial and ethnic disparities remain an issue in HIV care in the United States, where black and Latino patients have lower rates of retention in care and viral suppression than white patients.^9^ Discontinuity of care is higher for male than female patients and higher for black than non-black patients.^10^ Nonwhite race is a risk factor for poor ART adherence, in addition to psychosocial factors of self-efficacy, depression, stigma, and stressful life events.^2^

Despite many barriers to retention in care, interventions to improve engagement have shown promise. For example, enhanced contact by phone between clinic visits and involvement of peer mentors or coaches can reduce rates of missed appointments.^11,12^ In recent years, the potential for technology to support retention in care has gained attention. PLWH increasingly turn to online sources for information and support.^13–15^ In addition, smartphone use continues to expand, even among patients of lower socioeconomic status, providing opportunities for tailored outreach and communication outside of clinical settings.^16^ Patients express interest in mobile health strategies that are engaging and useful, but have concerns about security and privacy, particularly in the context of HIV and substance use.^17–20^ Among young people with HIV, desired features of mobile health applications include the ability to connect to a community of other PLWH, connect with healthcare providers, track personal data, and obtain news and education about their health.^21^

Mobile phone-based interventions can improve patient self-management and adherence in chronic disease.^22^ However, evidence for effectiveness has been mixed, with mostly small-scale studies and heterogeneous study designs.^23^ Mobile phone features can include medication alerts, refill, and appointment reminders; tracking of clinical data; access to resources; messaging functions; and social networking.^24^ In HIV care, online support groups can promote risk reduction and psychological health for PLWH.^25^ Text messaging interventions can improve adherence to ART, reduce nonattendance at clinic appointments, and improve physiological measures of CD4 counts and viral loads.^26–28^ Mobile health interventions that are based on smartphone applications (apps) have some advantages over texting, which include richer functionality and enhanced security. HIV apps currently commercially available are not rigorously evidence based and lack many of the features that patients endorse as important to them.^29^ For HIV mobile health interventions in recent development for research, greater emphasis is being placed on user-centered and theory-based design to guide tailoring to users' motivations and preferences.^30^ Further work is needed to create mobile health interventions that address retention in HIV care, target vulnerable populations, and evaluate long-term outcomes while maximizing potential benefits of mobile health and mitigating its risks.^31–34^

With these parameters in mind, we designed and piloted a smartphone-based intervention, PositiveLinks (PL), to promote linkage to and engagement with HIV care, and to improve clinical outcomes. We targeted the rural southern United States, where disparities in HIV care disproportionately affect vulnerable populations who face racial inequality, poverty, stigma, trauma, lack of social support, and substance use.^35–39^ To our knowledge, PL is unique as a clinic-based mobile intervention to connect patients to care and to a virtual local community, with sustained engagement over a 12-month period and assessment of longitudinal clinical outcomes. Our primary hypotheses were that patients in the PL program would have higher retention in care at 6- and 12-month follow-up than at baseline as well as higher CD4 counts and a higher likelihood of achieving a suppressed viral load at 6- and 12-month follow-up than at baseline. We also aimed to test exploratory hypotheses that patients who used the PL app more frequently would have higher retention in care than those who used it less frequently.

## Methods

### Design and setting

PositiveLinks was developed as a multicomponent intervention intended to improve linkage and retention in care for PLWH based at the University of Virginia Ryan White Clinic, an academic outpatient clinic serving a primarily nonurban patient population covering 52 counties in Virginia. This pilot study used a single-arm prospective design with baseline, 6-, and 12-month assessments.

### Recruitment of participants

Participants were HIV-positive adults recruited from the Ryan White Clinic, area AIDS service organizations (ASOs), and HIV testing sites. Participants were all receiving care from a single site (the Ryan White Clinic at UVA) or, if recruited from the community, initiating or restarting care at this site. Patients were eligible if they were newly diagnosed with HIV (within 90 days of study enrollment), returning to care after a lapse, or at risk of falling out of care as determined by their care provider. Providers assessed risk of falling out of care based on their experience with patients' missed appointments, prior difficulties with adherence, and psychosocial barriers to retention in care. Providers referred patients to the program by contacting the study team, who then contacted patients to assess eligibility. To be enrolled, patients had to score 40 or above on the Wide Range Achievement Test (WRAT-4) or pass a subsequent reading test, corresponding to approximately fourth grade reading level.^40^ The app design was tailored to accommodate low literacy. Patients provided written informed consent to participate in the study. IRB approval was obtained for the study. Enrollment occurred on a rolling basis between September 2013 and May 2015.

### Baseline data collection

During enrollment, patients consented to participate, completed the WRAT-4 literacy test, answered baseline questions, and learned how to use the study smartphone and PL app. Participants' demographic characteristics included age, gender, race, transmission risk behavior, and time since diagnosis. Socioeconomic variables included education, self-reported income, and distance traveled to clinic. Clinical data and appointment data were abstracted from the electronic medical record.

### PositiveLinks intervention

The PL intervention included a custom smartphone app that was developed through an iterative, user-centered process with patients at the Ryan White Clinic in formative interviews and subsequent user testing. The development was informed by our team's prior work on text-based mobile interventions, which demonstrated that PLWH would respond to bidirectional queries and valued tailoring of messages to their responses.^41,42^ Development was also informed by the emerging literature on preferences of PLWH regarding mobile health interventions.^18–21^ Additional formative work with our target users included interviews to identify desired features and the frequency and customization of queries.^43–45^ Participants in the formative phase expressed interest in app features that would promote self-monitoring, offer HIV-related and health-related information, and facilitate interaction with other PLWH. They were able to beta test early versions of the app to give feedback on the usability and further tailoring.

**FIG. 1.**
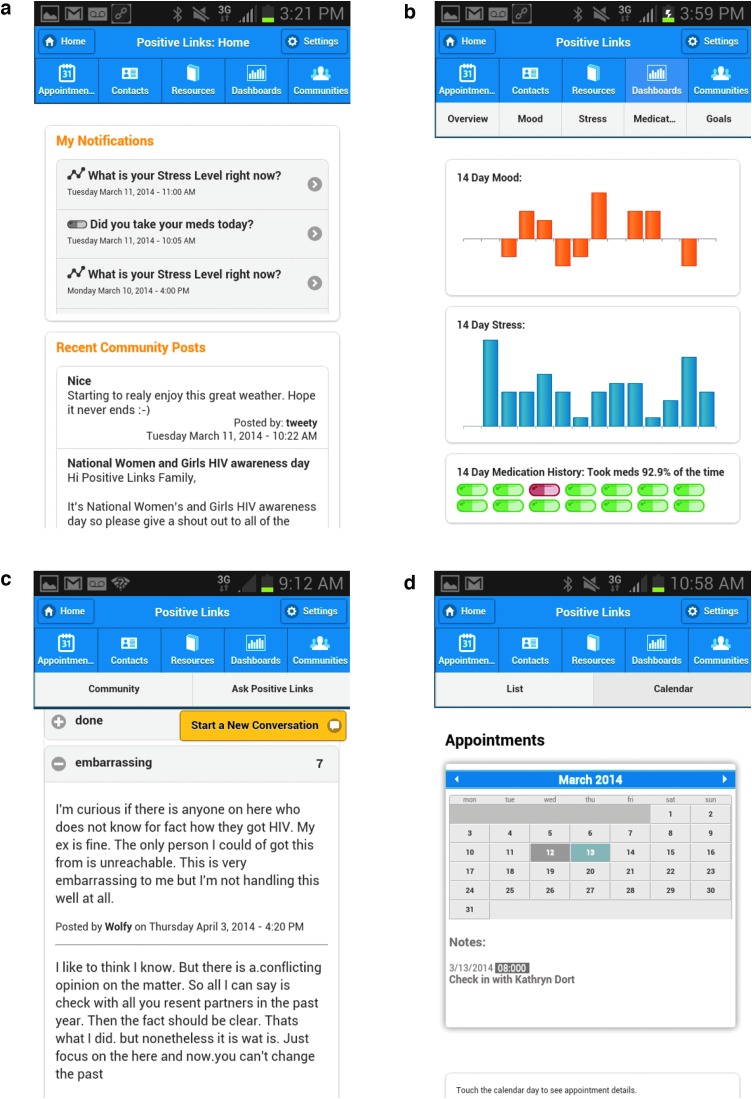
Example images of the Positive Links app: **(a)** app home screen; **(b)** member dashboard overview; **(c)** community message board; **(d)** appointment page.

The app included the following features: tailored educational resources; daily queries of stress, mood, and medication adherence; weekly quizzes; appointment reminders; and a community message board (CMB). Figure 1 shows screenshots of the app features. The educational resources included information that had been requested by patients involved in the formative phase, such as orientation to the clinic, HIV-related and health information, and stress reduction techniques. For the CMB, participants selected user names to protect anonymity and could start new conversations on the board or respond to older conversations. The PL team intermittently introduced new conversation topics on HIV or general well-being. The team monitored the board for misinformation, identity disclosure, and inflammatory comments. The team could communicate with participants privately, as needed, to address technical issues and assist with care coordination. Contact information for the clinic-affiliated PL team was included in the app and participants could reach them by phone call or text. Participants' app data, including query responses and CMB posts, were accessible to the PL team through an administrative web portal.

At the time of enrollment, participants were given Samsung Galaxy 2 or Galaxy 3 smartphones with the PL app installed and a voice/data plan with unlimited minutes, texting, and data for the 12-month study period. The PL app was a native app installed on the study phones and designed for their Android operating system, not a hybrid or WebApp. Phones were encrypted and password protected and had a remote locate and wipe functionality. The app was also password secured.

### Outcomes

The two primary outcomes were retention in care and visit constancy. Participants were categorized as retained in care if they kept 2 appointments with an HIV care provider that were separated by 90 days within a 1-year period (HRSA-1 definition).^46^ Visit constancy is the proportion of 4-month time intervals in which 1 visit with an HIV care provider was completed in a 1-year time period. Participants were given a score of 3, 2, 1, or 0 if they had kept appointments in 3, 2, 1, or 0 of the time intervals, respectively. The two secondary clinical outcomes were CD4 count (cells/mm^3^) and HIV viral load (copies/mL), extracted from the electronic medical record. Viral load values are represented as log10(1+VL) and as proportion of participants with suppressed viral load (VL<200). HIV care guidelines through the duration of the study recommended a clinic visit and laboratory testing every 3 months.

### Data collection

Participants completed usability interviews after 3 weeks of enrollment, so the team could address any technical difficulties and obtain feedback on use of the app. Follow-up assessments were completed at 6 and 12 months after enrollment. Google Analytics was used to evaluate the cohort's usage of the app, measured by app launches. The study team monitored response rates to queries and quizzes, as well as CMB posts, through the PL administrator web portal linked to the app. Data were reviewed by the PL team at weekly operational meetings. App usage reported is cumulative starting at enrollment. Participants received $25 gift cards for completion of the 12-month assessment. CD4 counts and viral loads were not collected by the study, but extracted from the participants' medical records. Laboratory results performed within 90 days before or after participants' 6-month and 12-month assessments were included.

### Statistical analyses

Descriptive statistics were calculated to evaluate baseline characteristics and frequency of use for app features. Outcomes were analyzed using an intention-to-treat approach. Retention in care was analyzed using McNemar's tests to assess for changes in participants' HRSA-1 status and proportion of participants with a visit constancy level >2. Clinical outcomes were analyzed using nonparametric Wilcoxon signed-rank tests to assess for changes in CD4 counts and HIV viral loads. Percentage of viral suppression was assessed with McNemar's test. Differences in outcomes by app use were assessed using Kruskal–Wallis tests. To assess for differences in outcomes by demographic or socioeconomic variables, multivariable analyses were performed using generalized estimating equations (GEE) to account for multiple assessments performed on each participant. Analyses were performed using SAS 9.4 and GAUSS 16.0.

## Results

### Participant characteristics

Of the 111 patients approached, 87 were eligible for recruitment and of these, 77 enrolled in PositiveLinks. During the study follow-up, two participants dropped out of the study (at week 30 and week 31) and one participant died (at week 9). Three participants were lost to follow-up of their phone data after 20, 25, and 37 weeks. CD4 counts and viral load data were obtained from the medical records for all 77 participants at baseline, 67 participants at 6 months, and from 61 participants at 12 months. Three participants were missing 6-month CD4 and viral load data, but had 12-month CD4 and viral loads available. All participants had appointment data at all time points, obtained from the medical records.

Participants' baseline characteristics are shown in Table 1. Of the 77 participants, 64% were male, 49% black non-Hispanic, 34% white non-Hispanic, and 8% Hispanic. More than half of participants reported incomes below 50% of the federal poverty level and a quarter had unstable housing status. Participants traveled an average of 37 miles and 47 min to the HIV clinic, with a maximum of 200 miles and 127 min. Self-reported transportation costs to travel to care visits averaged $11 (SD $15) with maximum up to $80.

**Table 1.** Participant Characteristics

	*Total (*N = *77)*
Gender, *n* (%)	
Male	49 (64)
Female	26 (34)
Transgender male to female	2 (3)
Race/ethnicity, *n* (%)	
White non-Hispanic	26 (34)
Black non-Hispanic	38 (49)
Hispanic	6 (8)
Asian	1 (1)
Multiple races	5 (6)
Refused	1 (1)
Income compared with federal poverty level (FPL), *n* (%)	
0% ≤ FPL <50%	45 (58)
50% ≤ FPL <100%	11 (14)
100% ≤ FPL <150%	12 (16)
150% ≤ FPL <200%	5 (6)
200% ≤ FPL	4 (5)
Risk factor, *n* (%)	
Heterosexual	37 (48)
Injection drug use (IDU)	3 (4)
IDU/MSM	2 (3)
Men who have sex with men (MSM)	31 (40)
Transgender male to female	2 (3)
Do not know/missing	2 (3)
Level of education, *n* (%)	
≤6 Years	1 (1)
7–11 Years	14 (18)
High school graduate	27 (35)
General equivalency diploma (GED)	8 (10)
Community college	2 (3)
Trade or technical school	4 (5)
Some college	15 (19)
College graduate	6 (8)
Baseline HRSA-1, *n* (%)	
No	38 (49)
Yes	39 (51)
Baseline visit constancy, *n* (%)	
1	42 (55)
2	18 (23)
3	17 (22)
Distance in miles from clinic, mean (SD)	37 (37)
Travel time in minutes to Clinic, mean (SD)	47 (31)
Housing status, *n* (%)	
Own place, room, apartment, or house	57 (74)
Temporarily doubled up with others	15 (19)
Temporary, transitional housing program	1 (1)
In shelter for homeless people	2 (3)
On the street or outside	1 (1)
Someplace else	1 (1)
Time in months living in present home situation, mean (SD)	41 (74)
Enrollment characteristics, mean (SD)	
Months from HIV diagnosis to enrollment	60 (76)
Age in years at enrollment	36 (12)
Clinical characteristics, mean (SD)	
Baseline CD4^+^	522 (373)
Baseline VL	23,682 (60820)
Baseline log10(1+VL)	2.46 (1.79)
Baseline appointment Adherence	85 (23)

### App usage

Table 2 shows usage of app features by participants. Mean response rates to daily queries assessing medication adherence, mood, and stress levels were 50%, 47%, and 47%, respectively, at 6 months and 41%, 39%, and 39%, respectively, at 12 months. Mean response rates to weekly quizzes assessing general and HIV-specific knowledge were 43% at 6 months and 37% at 12 months. Posts per participant to the CMB showed a mean of 12.2 posts (SD 22) at 6 months and 19 posts (SD 36.4) at 12 months. Mean total app launches per participant were 188 (SD 183) at 6 months and 312 (SD 338) at 12 months. Overall, some reduction in query response rates occurred over time, but almost 40% of participants were still actively using the app after 1 year, as defined by query responses.

**Table 2.** Participants' App Utilization

	*6 Months*		*12 Months*^a^	
*PL participant app utilization*	*Mean (*n* = 77)*	*SD*	*Mean (*n* = 77)*	*SD*
Medication query response rate	50%	36%	41%	35%
Mood query response rate	47%	36%	39%	35%
Stress query response rate	47%	36%	39%	35%
Quiz response rate	43%	34%	37%	34%
Total CMB posts	12.2	22.0	19.0	36.4
Total app launches	188	183	312	338

^a^ Data at 12 months are cumulative and includes 6 months' data.

CMB, community message board.

### Outcome measures

At baseline, 51% met the HRSA-1 definition of retention in care. At 6 months, 88% were retained in care, a significant improvement from baseline (*p* < 0.0001, McNemar's test) and 81% at 12 months, also a significant improvement from baseline (*p* = 0.0003, McNemar's test). At baseline, 22% of participants had the highest visit constancy (kept appointments in all three time intervals). At 6 months, 36% had the highest visit constancy, a significant increase (*p* = 0.0164, McNemar's test) and at 12 months, 51% had the highest visit constancy, also a significant increase (*p* = 0.0004, McNemar's test).

**FIG. 2.**
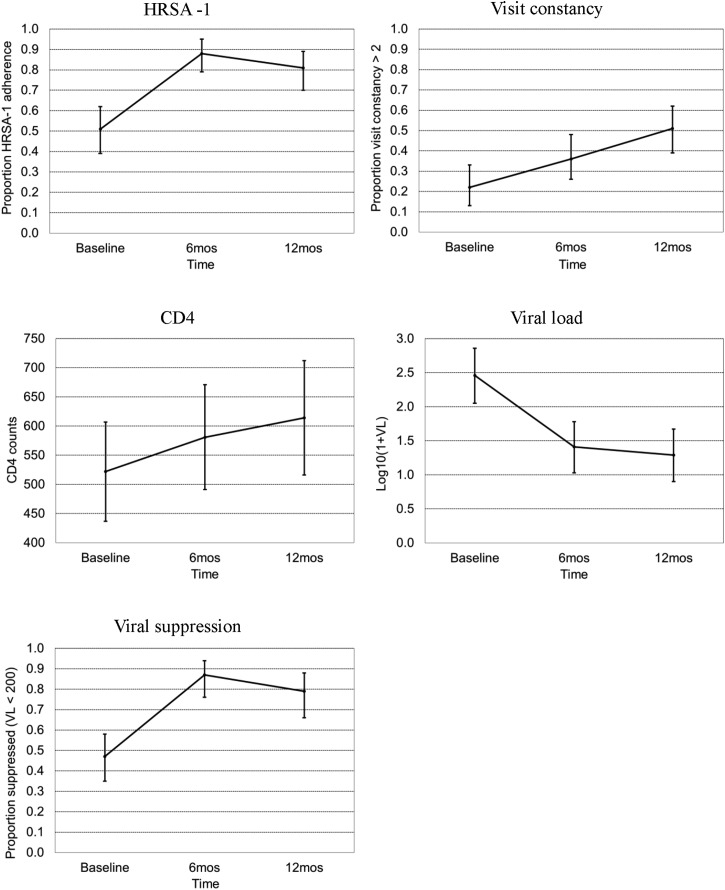
Summary of outcomes at baseline, and 6- and 12-month follow-ups.

Participants' mean CD4 counts increased from 522 cells/mm^3^ (SD 373, *n* = 77) at baseline to 581 (SD 369, *n* = 67) at 6 months (*p* = 0.0007) and 614 (SD 383, *n* = 61) at 12 months (*p* = 0.0005, Wilcoxon signed-rank test). Participants' mean viral loads decreased from 23,682 copies/mL (SD 60821, *n* = 77) at baseline to 14,912 (SD 48530, *n* = 67) at 6 months (*p* = 0.0023) and 13,890 (SD 84919, *n* = 61) at 12 months (*p* = 0.0073, Wilcoxon signed-rank test). These results are shown in Table 3 and displayed visually in Fig. 2.

**Table 3.** Changes in HRSA-1, Visit Constancy, CD4 Counts, and Viral Loads at Baseline, 6, and 12 Months

	*Baseline*	*6 months*		*12 months*	
*HRSA-1 compliance*^a^	*% (95% CI)*	*% (95% CI)*	p *Value vs. baseline*	*% (95% CI)*	p *Value vs. baseline*
	51 (39, 62)	88 (79, 95)	<0.0001	81 (70, 89)	0.0003
Visit constancy^a^	% (95% CI)	% (95% CI)		% (95% CI)	
Percent >2	22 (13, 33)	36 (26, 48)	0.0164	51 (39, 62)	0.0004
CD4 counts^b^	Mean (95% CI), *n* = 77	Mean (95% CI), *n* = 67		Mean (95% CI), *n* = 61	
	522 (437, 607)	581 (491, 671)	0.0007	614 (516, 712)	0.0005
VL suppressed (VL <200)^a^	% (95% CI), *n* = 77	% (95% CI), *n* = 67		% (95% CI), *n* = 61	
	47 (35, 58)	87 (76, 94)	<0.0001	79 (66, 88)	0.0007
Log10(1+VL)^b^	Mean (95% CI), *n* = 77	Mean (95% CI), *n* = 67		Mean (95% CI), *n* = 61	
	2.46 (2.05, 2.86)	1.41 (1.03, 1.78)	<0.0001	1.29 (0.90, 1.67)	0.0012

^a^ *p* Value from McNemar's test.

^b^ *p* Value from Wilcoxon signed-rank test.

For comparison of viral load outcomes, we assessed laboratory data from the patient population receiving care at the Ryan White clinic during the time period covered by the PL intervention. These data were deidentified and obtained in aggregate from clinic reporting for 730 patients. Viral suppression was defined as less than 200 copies. In 2014, 88.9% of patients at the clinic had suppressed viral loads, increasing to 90.0% in 2015 and 90.9% in 2016. The PL program specifically targeted patients who were new to care or at risk of falling out of care as determined by their HIV care providers. For participants in PL, 47% (95% CI 35–58%, *n* = 77) of participants had viral suppression at baseline, which increased significantly to 87% (76–94%, *n* = 67) at 6 months (*p* < 0.0001) and 79% (66–88%, *n* = 61) at 12 months (*p* = 0.0007).

### Outcomes by app usage

Higher app use occurred among those who were retained in care both at baseline and 6 months. The highest app use was observed among those not classified as retained in care by HRSA-1 at baseline, but who were retained in care at 6 months. In contrast, participants who did not meet HRSA-1 measures for retention in care at 6 months, had the lowest mean app launches, query response, CMB posts, and quiz responses in the cohort (Table 4). However, these trends were not statistically significant (*p*-values from Kruskal–Wallis tests). A similar pattern was seen at 12 months with higher app use among those consistently retained in care versus those consistently out of care. The highest app use was again observed among those who changed from out of care to retained in care. The difference in CMB posts (*p* = 0.035, Kruskal–Wallis test) was significantly different between groups during this time period.

**Table 4.** Participants' App Utilization By HRSA-1 at Baseline, 6, and 12 Months

*App use at 6 months by HRSA-1 at baseline and 6 months*	*Baseline = No* *6 months = No (*n* = 7) Mean (SD)*	*Baseline = Yes* *6 months = No (*n* = 2) Mean (SD)*	*Baseline = No* *6 months = Yes (*n* = 31) Mean (SD)*	*Baseline = Yes* *6 months = Yes (*n* = 37) Mean (SD)*	p* Value*^a^
Med query response rate	32 (24)	23 (32)	59 (36)	47 (36)	0.171
Mood query response rate	29 (24)	24 (32)	58 (35)	42 (36)	0.141
Stress query response rate	28 (23)	22 (30)	58 (35)	42 (36)	0.128
Quiz response rate	21 (23)	19 (27)	54 (33)	40 (34)	0.083
Total CMB posts	7.4 (8.2)	9.0 (12.7)	19.9 (30.5)	6.9 (12.2)	0.105
Total app launches	129 (74)	62 (62)	256 (204)	149 (167)	0.087

*App use at 12 months by HRSA-1 at baseline and 12 months*	*Baseline = No* *12 months = No (*n* = 6) Mean (SD)*	*Baseline = Yes* *12 months = No (*n* = 9) Mean (SD)*	*Baseline = No* *12 months = Yes (*n* = 32) Mean (SD)*	*Baseline = Yes 12 months = Yes (*n* = 30)*	p* Value*^a^
Med query response rate	38 (31)	19 (31)	47 (37)	41 (35)	0.215
Mood query response rate	36 (30)	18 (31)	45 (36)	38 (35)	0.186
Stress query response rate	36 (30)	18 (31)	46 (36)	38 (34)	0.148
Quiz response rate	37 (32)	18 (29)	44 (34)	36 (33)	0.291
Total CMB posts	12.7 (12.3)	2.9 (6.0)	28.3 (48.4)	15.0 (27.0)	0.035
Total app launches	326 (299)	138 (235)	381 (359)	289 (342)	0.087

^a^ *p* Value from Kruskal–Wallis test comparing all four groups

CMB, community message board; Med, medication.

Multivariate analyses investigated whether socioeconomic status or demographic factors, including age, race, sex, income, education, and time from HIV diagnosis to enrollment were predictive of the changes observed in HRSA-1, CD4 counts, or viral loads. GEE were used to account for multiple assessments on each subject. The effect of each variable was assessed individually when added to a model that included an effect for follow-up. In each of the models, HRSA-1 achievement was significantly greater at 6 months than at baseline, and at 12 months than at baseline. No significant associations were identified between demographic factors and changes in the outcomes of HRSA-1, CD4 counts, or viral loads.

There were 17 participants diagnosed with HIV within 90 or fewer days from enrollment. As they were new to care, their HRSA-1 and visit constancy could be falsely low because of insufficient follow-up time. Therefore, analyses were repeated using only the 60 participants who had been in care for more than 90 days to assess changes in HRSA-1, visit constancy, CD4 counts, and viral loads; and participants' app utilization by HRSA-1 at baseline, 6 months, and 12 months. There were no significant differences in the findings of these analyses and the full data set of 77 participants.

## Discussion

This study showed that the PositiveLinks intervention, which includes a clinic-affiliated custom app that provided resources to patients and assisted in care coordination by clinic staff, resulted in improved retention in care at 6 and 12 months. CD4 counts and viral loads also improved at 6 and 12 months, perhaps due to improved retention in care for a population specifically targeted as high risk for poor retention. To our knowledge, this is the first published study of a clinic-affiliated smartphone app intervention for PLWH to demonstrate clear improvements in retention in care. These findings support the study hypotheses that the PL intervention would result in improved engagement in care and clinical outcomes at 6 and 12 months of follow-up. This is also the first study to demonstrate improvements in long-term HIV clinical outcomes for a custom smartphone app for PLWH.

Achieving sustained usage of mobile health tools can be a challenge.^22,24^ Participants showed sustained usage of the PL app, with 40% continuing to respond to daily queries of medication adherence, mood, and stress after 12 months. Through the app, participants also had access to the CMB, educational resources, appointment tracking, and messaging with the program coordinator. Several factors likely contributed to participants' persistent use. Fresh content was added throughout the 12-month period with new quizzes, resources, and discussion topics on the CMB. Although not directly incentivized for app use, participants who responded to 100% of the queries they received were entered into a monthly raffle and could win a $50 gift certificate. The qualitative analyses of the PL CMB and participant interviews indicate that the app provided a sense of connection and social support, which was highly valued.^47^ Participants reported that the app helped them in overcoming social and geographic isolation that had been barriers to accessing HIV care.^48^ The involvement of our patients at all steps in app development, from the formative phase to testing and piloting, likely also improved the fit of the program to their lifestyles and needs.

An important factor in the popularity and success of the PL intervention may be its underlying principle of “warm technology”.^49^ “Cold technology” is impersonal, isolating, and not concerned with feelings or emotion. In contrast, “warm technology” is personal, facilitates human contact, and shares emotions. The PL intervention was not just a stand-alone app, but a pathway to human contact. The program coordinator provided outreach and assistance to those who were having trouble with the phone or app or who reported issues with medication adherence, mood, or stress. The CMB allowed participants to connect with peers living with HIV who could give support, advice, and encouragement. The majority of prior mobile health interventions in HIV care have been focused primarily on alerts or reminders.^24^ While this component appears to be important in addressing adherence, it may not be sufficient. Text messaging interventions with bidirectional interactions and program coordinator support have shown some success in improving clinical outcomes.^27^ The concept of warm technology emphasizes human connection and allowed the PL intervention to take mobile strategies a step further, enhancing patients' relationships with their care setting and virtual community.

Although most prior Internet-based health interventions for PLWH in high-income countries have included predominantly white and educated populations, PL was developed for the rural United States. The majority of our participants self-identified as black and reported incomes below 100% of the federal poverty level. In the US South, low-income African American PLWH suffer worse HIV care outcomes.^36–38^ This program eliminated that disparity, as the portion of the cohort that self-identified as black, non-Hispanic had retention in care that was not significantly different from those who self-identified as white. The PL program was successfully deployed to the demographic group who may need it most.

In this study, usage appears to be related to outcomes, with those who used the app the most demonstrating the greatest improvement in outcomes. At both 6 and 12 months, a similar pattern appeared: those who made no improvement, or regressed, had the lowest app usage across all categories of app interaction. In contrast, those who achieved HRSA-1 retention measures at baseline and maintained this at follow-up used the mobile platform at the second highest level. Those who did not achieve retention at baseline but transitioned to meeting HRSA-1 retention measures at follow-up used the app the most. This change was statistically significant only for the number of CMB posts at 12 months. However, a limitation of this pilot study was that it was underpowered to detect relationships between overall app use or specific app features and retention in care or clinical outcomes.

Additionally, limitations include the fact that this study was a single-arm prospective pilot study with a relatively small sample size. These factors limit our ability to attribute outcomes to the intervention rather than to uncontrolled other factors. We did compare viral suppression rates among PL participants to the clinic as a whole. Overall, viral suppression improved for the clinic population, likely due to many factors, including the efforts of retention-in-care staff, case managers, peer coaches, and other support available for our patients. PL specifically targeted patients at risk of poor outcomes and enrolled patients who had baseline rates of viral suppression much lower than the clinic average. These high-risk patients achieved significant improvements in viral suppression, bringing them almost up to the rates of the clinic as a whole. These results point to a benefit to the intervention, even though the lack of a randomized control group makes it challenging to infer causation.

It is possible that retention in care outcomes may be falsely low for participants who are new to care, due to insufficient follow-up time. However, restricting the analysis to participants with longer follow-up time did not significantly change the results or conclusions. It is also important to note that study phones were given to participants with the app installed. Some of the intervention's appeal, especially to participants living in poverty, may be the phone itself, independent of the app. Finally, the app is affiliated with the clinic, which may increase its impact but could limit generalizability to other populations or contexts.

This intervention builds on emerging evidence that mobile health strategies can engage patients with chronic diseases, and in particular, PLWH. Prior text messaging-based interventions for PLWH have provided self-management, reminders, and access to assistance.^44,45^ Moving from SMS texting to app development allows increased functionality and improved security, including HIPAA compliance, which can be of particular concern in the context of HIV and for those who report substance use.^34^ The PL intervention addresses these needs and incorporated modifications for low literacy into its design, aiming to be accessible to vulnerable patients. The findings support further development and dissemination of clinic-affiliated mobile health interventions to improve retention in HIV care. Successful program implementation may require that healthcare systems invest in phones and phone plans for disadvantaged patients.

In conclusion, mobile health interventions have the potential to improve retention in care and clinical outcomes for PLWH. Long-term benefits can be achieved through a smartphone app connected to a clinical care setting and can reach vulnerable patients. Next steps for this project include installation of the app on users' own phones (if they already possess one) and subsidies for phones and service (if needed) to make the app accessible to more users. Further investigation of subgroup usage patterns may point to refining features that will encourage ongoing use by more participants. Integration of PositiveLinks into clinical care is planned, along with extension to other HIV care sites.
